# Intraocular pressure changes and pain scores within 24 hours and short-term outcomes after micropulse transscleral laser therapy

**DOI:** 10.1371/journal.pone.0340625

**Published:** 2026-02-06

**Authors:** Nattanan Phanvichatkul, Pukkapol Suvannachart

**Affiliations:** 1 Department of Ophthalmology, Suddhavej Hospital, Mahasarakham University, Mahasarakham, Thailand; 2 KKU Glaucoma Center of Excellence, Department of Ophthalmology, Faculty of Medicine, Khon Kaen University, Khon Kaen, Thailand; Alexandria University Faculty of Medicine, EGYPT

## Abstract

**Purpose:**

To assess intraocular pressure (IOP) changes and pain scores during 24 hours and short-term outcomes after micropulse transscleral laser therapy (MP-TLT).

**Design:**

Retrospective case series.

**Methods:**

We reviewed eyes undergoing MP-TLT (2,000 mW; 31.3% duty cycle; 100–200 seconds) with serial IOP measurements during the first operative day, excluding those with prior cyclophotocoagulation or combined procedures. IOP and pain scores (numerical rating scale, NRS) were recorded at 1, 5, 9, 13 hours, and the following day post-procedure. Data from follow-up visits were obtained to evaluate success rates (≥30% IOP reduction or an IOP 6–18 mmHg). Mixed-effects regression and Kaplan–Meier method were used for analysis.

**Results:**

This study examines 46 eyes from 40 patients, 58.7% with secondary glaucoma. The mean preoperative IOP was 40.6 mmHg. The NRS was 2.3 at the end of the procedure. The mean postoperative IOP (% reduction) and NRS values were 33.9 (16.5) mmHg and 2.4 at 1 hour, 36.1 (11.1) mmHg and 2.8 at 5 hours, 32.6 (19.7) mmHg and 1.7 at 9 hours, 29.7 mmHg (26.8) and 1.2 at 13 hours, and 24.5 (39.7) mmHg and 1.0 on the following day. The IOPs were significantly lower at all time points (p < 0.05), with the lowest mean value of 16.4 mmHg at week 1. The success rate was 80.6% at 12 months. One patient developed transient hypotony maculopathy.

**Conclusions:**

MP-TLT resulted in a significant IOP reduction within 24 hours, with maximal effect at 1 week, and demonstrated favorable short-term outcomes.

## Introduction

Micropulse transscleral laser therapy (MP-TLT) represents an approach of transscleral cyclophotocoagulation, designed to minimize damage to the ciliary body. By delivering laser energy in short bursts, MP-TLT allows tissue to cool between pulses, thereby reducing thermal damage and inflammation. The mechanisms of action for MP-TLT include reducing aqueous humor production through selective destruction of the ciliary processes and increasing uveoscleral outflow [1,2].

MP-TLT is an increasingly used non-incisional procedure that has been reported to be effective in reducing intraocular pressure (IOP) for various types of glaucoma, including primary and secondary causes, as well as stages of the disease, from mild to refractory cases in both children and adults [3–5]. Previous studies have reported promising results with MP-TLT, showing outcomes comparable to continuous wave transscleral cyclophotocoagulation in terms of IOP reduction, with lower rates of serious ocular complications [6–8]. The efficacy of MP-TLT varies according to different factors, including laser parameters, techniques, initial IOP, and types of glaucoma diagnosis [1,9].

While the intermediate to long-term efficacy of MP-TLT has been well-documented, including reports of IOP reduction on the first postoperative day [6,10,11], there is limited research on how IOP changes within the first 24 hours following the procedure. Early IOP control is crucial in glaucoma patients, especially those with advanced disease. Therefore, this study primarily aimed to investigate the effect of MP-TLT on IOP, along with pain scores, during the first 24 hours post-treatment. Short-term IOP outcomes were evaluated as a secondary objective.

## Methods

This retrospective case series included glaucoma patients who were treated with MP-TLT from March 2021 to June 2023 at Suddhavej Hospital, Mahasarakham University, Thailand. The study followed the tenets of the Declaration of Helsinki and was approved by the Institutional Review Board of Mahasarakham University (276–284/2565). The requirement for written informed consent was waived due to the retrospective nature of the study. The inclusion criteria consisted of patients diagnosed with glaucoma who received MP-TLT and had serial IOP measurements during the first operative day. Patients with a history of cyclophotocoagulation by any method, or the presence of adjunctive procedures, e.g., anterior chamber irrigation and intravitreal injection, were excluded. The authors accessed the data after obtaining Institutional Review Board approval. Data collection was conducted between 1 September 2023 and 30 November 2023. All patient information was handled in a deidentified format to ensure confidentiality.

### Surgical procedure

All procedures were performed by a glaucoma surgeon, primarily under local anesthesia, including sub-Tenon, retrobulbar and peribulbar injections with 2–3 ml of 2% lidocaine. General anesthesia was reserved for uncooperative patients. MP-TLT was conducted using the Vitra 810 and Subcyclo probe (Quantel Medical, France) with the following settings: a laser power of 2,000 mW, duty cycle of 31.3%. The standard treatment protocol consisted of 200 seconds over 360° of the limbal circumference. Treatment parameters were individualized in selected cases; eyes with thin sclera, prior trabeculectomy, or an existing glaucoma drainage device received reduced treatment arcs or shorter durations at the surgeon’s discretion. The probe was held perpendicular to the sclera at the ciliary body site as determined by the scleral transillumination technique in some cases or 3 mm from the limbus in other cases. A continuous back-and-forth motion with a sweeping velocity of 10 seconds per hemisphere was used, avoiding regions of scleral thinning with 0.2% carbomer eye gel as a contact medium.

At the end of the procedure, patients receiving local anesthesia were asked to rate their pain using a numerical pain rating scale (NRS) from 0 to 10. Postoperatively, patients received a combined solution of 0.5% neomycin sulfate and 0.1% dexamethasone in eye drops four times daily, along with all pre-existing antiglaucoma medications and paracetamol (500 mg), one tablet as needed every six hours. No cycloplegic drops were prescribed. During admission after surgery, registered nurses evaluated IOP using the iCare IC200 rebound tonometer (Icare Finland Oy, Finland) and assessed pain scores using NRS at 1 hour, 5 hours, 9 hours, 13 hours, and 1 day before hospital discharge.

### Data collection and statistical analyses

We collected demographic data on age, gender, laterality, type of diagnosis, comorbidities, lens status, and history of previous ocular surgery. Preoperative data were collected from the clinic visit immediately preceding surgery, while postoperative data were gathered at the following intervals: within the first day, 1 week, 1 month, 3 months, 6 months, and 12 months. This data included best-corrected visual acuity (BCVA) measured by the Snellen chart, IOP, number of antiglaucoma medication classes, and pain scores measured by NRS. For IOP, measurements were taken using the iCare tonometer during admission on the first postoperative day, and with either the Goldmann applanation tonometer or iCare during clinic visits. A total of seven antiglaucoma medication classes included topical beta-blockers, topical alpha-adrenergic agonists, topical or oral carbonic anhydrase inhibitors, topical prostaglandin analogs, topical Rho-kinase inhibitors, topical cholinergic agonists, and hyperosmotic agents. Pain was also presented using Jensen’s classification, including no pain (NRS 0), mild pain (NRS 1–4), moderate pain (NRS 5–6), and severe pain (NRS 7–10) [12].

Statistical analyses were performed using Stata 13.0 (Stata Corp, College Station, TX, USA). Demographic data were presented with appropriate descriptive statistics. BCVA data were converted and presented as logMAR values. The primary outcome of the IOP profile, along with pain scores, in the first 24 hours, were compared to preoperative values using mixed-effects regression. We also did a subgroup analysis by baseline IOP level and diagnosis since these are factors affecting MP-TLT efficacy. For comparisons between preoperative and final visits, paired t-tests and Wilcoxon’s matched-pairs tests were used for continuous variables, depending on their distribution, with McNemar’s test applied for categorical variables.

Secondary outcomes included short-term surgical success, assessed using the Kaplan–Meier method, and postoperative complications, such as a loss of ≥2 lines of BCVA on the Snellen chart, persistent anterior chamber inflammation at 3 months, cystoid macular edema, and hypotony (IOP ≤ 5 mmHg). For surgical success at 6 and 12 months, we used the success criteria defined as a ≥ 30% IOP reduction from preoperative values or an absolute IOP of 6–18 mmHg. Failure was defined as an IOP outside the specified criteria for two consecutive visits, sight-threatening complications, or requiring subsequent glaucoma surgery. A p-value <0.05 was deemed statistically significant.

## Results

This study included 46 eyes from 40 patients with males predominating (67.39%). The average age of the patients was 56.3 years. Most eyes had secondary glaucoma (58.7%) and were phakic (58.7%). The demographic data are presented in Table 1. MP-TLT was performed on four quadrants in 76.1% of cases, with a maximum total duration of 200 seconds in 67.4% of eyes, primarily under sub-Tenon anesthesia (76.1%). Scleral transillumination was performed on 18 eyes, with the mean (SD) distance from the limbus to the ciliary body being 2.72 (0.42) mm.

**Table 1:** Demographic Data.

	All
**Number of eyes (patients)**	46 (40)
**Age (patients, years)**	
Mean ± SD	56.3 ± 23.7
**Gender (patients, %)**	
Male	27 (67.5)
Female	13 (32.5)
**Laterality (eyes, %)**	
Right	24 (52.2)
Left	22 (47.8)
**Diagnosis (eyes, %)**	
Primary glaucoma	11 (23.9)
Primary open angle glaucoma	6 (13.0)
Primary angle closure glaucoma	5 (10.9)
Secondary glaucoma	27 (58.7)
Neovascular glaucoma	13 (28.3)
Traumatic glaucoma	6 (13.0)
Other secondary glaucoma	8 (17.4)
Childhood glaucoma	8 (17.4)
Juvenile open angle glaucoma	2 (4.4)
Primary congenital glaucoma	2 (4.4)
Secondary childhood glaucoma	4 (8.7)
**Lens status (eyes, %)**	
Phakic	27 (58.7)
Pseudophakic	16 (34.8)
Aphakic	3 (6.5)
**Comorbidities (eyes, %)**	
Hypertension	20 (43.5)
Diabetes mellitus	16 (34.8)
**Previous ocular surgery (eyes, %)**	
Cataract surgery	19 (41.3)
Trabeculectomy	4 (8.7)
Glaucoma drainage device implantation	3 (6.5)
Penetrating keratoplasty	4 (8.7)
Vitreoretinal surgery	4 (8.7)
None	12 (26.1)
**Indications for surgery (eyes, %)**	
Painful blind eyes or eyes with poor vision	18 (39.2)
Primary procedure in sighted eyes with favorable risk-benefit over incisional surgery	10 (21.7)
Primary procedure in eyes with poor visual potential	10 (21.7)
Failed filtering surgeries	8 (17.4)

SD indicates standard deviation.

**Fig 1:**
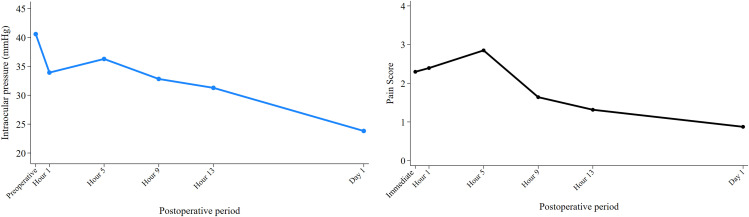
Linear plots of intraocular pressure and pain score changes within 24 hours. **A**, Intraocular pressures. **B**, Pain scores.

The median follow-up time was 5.2 months. The preoperative mean IOP was 40.6 mmHg, with an average of 3.9 antiglaucoma medication classes (Table 2). After treatment, the mean IOP decreased significantly at all postoperative time points, dropping to 33.9 (16.5% reduction), 36.1 (11.1%), 32.6 (19.7%), 29.7 (26.8%), and 24.5 (39.7%) mmHg at 1, 5, 9, 13 hours, and 1 day, respectively (Fig 1, Table 3). These reductions were statistically significant from the first postoperative hour onward (Table 3). The lowest mean IOP (±SD) was observed at the first-week visit, measuring 16.4 ± 10.9 mmHg (p < 0.001), followed by 23.5 ± 14.9 mmHg at 1 month (p < 0.001), 20.7 ± 12.4 mmHg at 3 months (p < 0.001), 20.2 ± 12.5 mmHg at 6 months (p < 0.001), and 19.0 ± 13.9 mmHg at 12 months (p < 0.001). At the final follow-up, the mean IOP (±SD) was 20.5 ± 13.9 mmHg (Table 2). The number of antiglaucoma medications decreased significantly to 3.4 classes (p = 0.019), while the use of systemic antiglaucoma medications reduced from 56.5% to 21.7%, though not statistically significant (Table 2). The success rates (95% CI) of MP-TLT were 86.0% (62.1, 95.3) and 80.6% (55.8, 92.3) at 6 and 12 months respectively. Ten eyes received additional glaucoma procedures, including repeat MP-TLT (6 eyes), and trabeculectomy (4 eyes).

**Table 2:** Clinical outcomes and treatment parameters.

	All (46 eyes)
**Follow-up duration**	
median, (IQR)	5.2 (0.2, 23.6)
**Anesthesia type (eyes, %)**	
Sub-Tenon	35 (76.1)
Retrobulbar/peribulbar	6 (13.0)
General	5 (10.9)
**MP-TLT treatment parameters**	
Area of treatment	
2 quadrants	8 (17.4)
3 quadrants	3 (6.5)
4 quadrants	35 (76.1)
Total duration (seconds)	
Minimum	100
Maximum	200
Mean (SD)	175 (40.1)
**Postoperative pain management (first 24 hours)**	
Number of eyes needed paracetamol (%)	16 (34.8)
Number of paracetamols needed (mean ± SD)	1.5 ± 0.7
Timing of paracetamol request (eyes, %)	
Hour 0–1	1 (2.2)
After hour 1–5	13 (28.3)
After hour 5–9	6 (13)
After hour 9–13	0
After hour 13-following day	2 (4.3)
**Best-corrected visual acuity in logMAR**	
Preoperative visit (median, IQR)	2.2 (0.7, 2.8)
Final visit (median, IQR)	2.3 (0.5, 3.5)
*p-value*	*0.266*
**Intraocular pressure (mean ± SD)**	
Preoperative visit	40.9 ± 17.2
Final visit	20.5 ± 13.9
*p-value*	<0.001*
**Number of antiglaucoma medications classes (mean ± SD)**	
Preoperative visit	3.9 ± 1.1
Final visit	3.4 ± 1.4
*p-value*	0.019*
**Use of systemic antiglaucoma medication (eyes, %)**	
Preoperative visit	26 (56.5)
Final visit	10 (21.7)
*p-value*	*0.14*
**Additional glaucoma procedure (eyes, %)**	
Repeat MP-TLT	6 (13.0)
Trabeculectomy	4 (8.7)

*p-value < 0.05

SD indicates standard deviation; IQR, interquartile range; MP-TLT, micropulse transscleral laser therapy.

**Table 3:** Comparison of intraocular pressure and pain during the first 24 hours postoperative period.

	Preoperative	Postoperative					
		Immediate	1 hour	5 hours	9 hours	13 hours	1 Day
**IOP (mmHg)**							
No. of eyes (%)	46 (100)	–	46 (100)	45 (97.8)	41 (89.1)	35 (76.1)	42 (91.3)
Mean (SD)	40.6 (17.2)	–	33.9 (17.0)	36.1 (17.7)	32.6 (16.4)	29.7 (15.9)	24.5 (14.5)
% reduction	reference	–	16.5	11.1	19.7	26.8	39.7
*p-value*	*reference*	–	0.001*	0.028*	<0.001*	<0.001*	<0.001*
**Pain**							
No. of eyes (%)		41 (89.1)	43 (93.5)	44 (95.7)	41 (89.1)	34 (73.9)	42 (91.3)
Severity (%)							
No pain	–	21 (45.7)	17 (40.0)	12 (26.1)	17 (40.0)	19 (41.3)	27 (58.7)
Mild	–	9 (19.6)	16 (34.8)	19 (41.3)	20 (43.5)	13 (28.3)	13 (28.3)
Moderate	–	6 (13.0)	8 (17.4)	9 (19.6)	4 (8.7)	2 (4.3)	2 (4.3)
Severe	–	5 (10.9)	2 (4.3)	4 (8.7)	0	0	0
Mean score (SD)	–	2.3 (2.9)	2.4 (2.5)	2.8 (2.3)	1.7 (1.7)	1.2 (1.6)	1.0 (1.5)
*p-value*	–	*reference*	*0.803*	*0.151*	*0.095*	0.018*	<0.001*

*P-value <0.05

IOP indicates intraocular pressure; SD, standard deviation.

All patients completed the laser treatment as planned, except for one who experienced severe pain under peribulbar anesthesia, limiting the treatment duration to 156 seconds. The immediate mean postoperative pain score was 2.3, which decreased over time, reaching statistical significance at postoperative hour 13 (Fig 1, Table 3). Patients experiencing immediate postoperative pain accounted for 54.3%, while 58.7–73.9% of patients experienced postoperative pain at any time point during the first operative day. Most patients reported mild pain at all time points, with the highest proportion (73.9%) reporting pain at 5 hours, which also had the highest mean pain score. None of the patients experienced severe pain after the ninth postoperative hour (Table 3). Paracetamol was needed in 34.8% of eyes, most frequently between hours 1 and 5 postoperatively, with no need for additional pain relief (Table 2).

Subgroup analysis by baseline IOP level showed that eyes with preoperative IOP > 40 mmHg exhibited a significant IOP reduction at all postoperative time points, whereas eyes with IOP ≤ 40 mmHg achieved significant reductions from 13 hours onward (S1 Table). At 5 hours postoperatively, both groups showed a mild transient rise in mean IOP accompanied by higher pain scores, although IOP levels remained below preoperative values (S1 Table). When analyzed by glaucoma diagnosis, a similar transient IOP and pain pattern was observed in the secondary glaucoma group (27 eyes), whereas the primary (11 eyes) and childhood (8 eyes) glaucoma groups demonstrated a continuous decline in IOP throughout the 24-hour period. All subgroups showed significant overall IOP reduction within 24 hours after MP-TLT (S1 Table).

Regarding complications, one eye experienced worsening of BCVA due to hypotony maculopathy following the procedure (200 seconds, four quadrants). This patient, diagnosed with advanced primary open-angle glaucoma, exhibited definite visual field progression despite low IOP (11–14 mmHg with four medication classes), which led to treatment with MP-TLT. At the one-week visit, IOP was 2 mmHg, and hypotony maculopathy with BCVA deterioration (20/70 to hand motion) was noted. Management involved discontinuing all antiglaucoma medications and observation, leading to gradual resolution between 8 and 11 months (BCVA 20/70 and IOP 6–9 mmHg without medication). No eyes developed cystoid macular edema, prolonged anterior chamber inflammation, or any other sight-threatening complications.

## Discussion

Our study demonstrated that the 24-hour serial IOP profile following MP-TLT showed a significant reduction starting from the first postoperative hour (16.5% reduction). Although a slight rise in IOP was observed at 5 hours, the mean IOP remained significantly lower than the preoperative value, with an 11.1% reduction. This contrasts with a recent report by Dervos et al., which observed significant IOP spikes within 6–12 hours post-procedure, with the mean IOP rising from 24.1 mmHg preoperatively to 31.7 mmHg [13]. In our subgroup with preoperative IOP ≤ 40 mmHg (mean 25.5 mmHg), no IOP spike was observed (S1 Table). This discrepancy may therefore be attributed to differences in patient characteristics and laser parameters. In their study, a higher total energy of 169 Joules was applied (3,000 mW power, 20-second sweep velocity per hemisphere, and 90-second duration per hemisphere), compared to a maximum of 125.2 Joules in our study, which represents a 25 percent difference in total energy. Notably, previous research has indicated an association between total energy exceeding 150 Joules and greater efficacy but also a higher risk of complications [14].

Fluence, the energy delivered per unit area, is another critical factor influencing IOP outcomes [15]. In addition to higher total energy, the 20-second sweep velocity per hemisphere resulted in approximately double the fluence used in our protocol [13]. Higher energy and fluence can cause more extensive damage to the ciliary body and adjacent tissues [16,17], leading to increased intraocular inflammation and potential IOP spikes, commonly found after continuous wave cyclophotocoagulation [18,19]. We hypothesize that balancing the total energy and fluence to achieve effective IOP reduction while minimizing inflammation can help reduce IOP spikes following MP-TLT. A recent consensus recommends a safe and effective MP-TLT setting of 2,500 mW power, 31.3% duty cycle, and four sweeps at a sweep velocity of 20 seconds per hemisphere, which is between the settings used in the study by Dervos et al and those used in our study [20].

Perioperative pain is another common concern with MP-TLT, ranging from 10.5% to 70% during the procedure [11,21,22], 83% within 12 hours postoperatively [21], and 5.8% to 18.4% on the following day [11,22]. Many factors are associated with pain in each patient, such as the type of anesthesia, surgical techniques, and individual pain thresholds, leading to variability in pain assessment across studies. In our study, most patients reported mild pain immediately after surgery (mean NRS = 2.3, mostly under sub-Tenon anesthesia), comparable to a similar population study (mean NRS = 3.57, under retrobulbar anesthesia with oral tramadol and diazepam) [21]. However, other studies have reported moderate pain levels, with mean NRS ranging from 5.86 to 6.02 [23,24]. Sukkee et al observed that some patients began to experience increased pain during the first 12 hours postoperatively [21], a trend also noted in our findings, particularly at the 5-hour mark. Interestingly, our study observed simultaneous elevations in both mean IOP and pain scores at this time point, suggesting a potential correlation between the two.

The mean IOP in our study was lowest at 1 week, consistent with previous studies [6,25,26]. However, other studies reported the lowest mean IOP at different times, such as at 1 month [27,28], likely due to the various factors mentioned earlier. The success rate of MP-TLT decreases over time and varies among studies due to factors such as treatment parameters and differing definitions of success [14]. The slight reduction in the number of antiglaucoma medications observed in our study is likely due to the discontinuation of systemic and some topical medications, particularly in cases of painful blind eyes where pain was controlled after the procedure, which represented the majority of the study population. Although our study demonstrated a favorable safety profile for most eyes, one patient developed hypotony maculopathy, which gradually resolved over 8 months, with best-corrected visual acuity returning to baseline (20/70). We suggest modifying treatment parameters, such as reducing the treatment area and duration, in similar cases of advanced disease with documented progression despite low preoperative IOP. Conversely, the treatment response may be insufficient in eyes requiring lower target IOPs. Despite favorable short-term success in our cohort, the mean final IOP of 20.5 mmHg remained above the defined success range (6–18 mmHg). The low rate of additional surgery likely reflects the predominance of eyes with markedly elevated preoperative IOP and poor visual potential, in which pain relief was the principal therapeutic goal.

The strength of our study is that we present detailed IOP and pain data at multiple time points within the first 24 hours after surgery, providing insights into their postoperative changes. IOP measurements during the first 24 hours were taken using the same device, ensuring consistency, although different personnel performed the measurements, and patient positioning was not controlled. The findings of our study may be applicable to clinical practice, as most patients undergo MP-TLT on an outpatient basis. Significant IOP spikes are less likely when using laser settings with comparable or lower total energy and fluence than those in our study, while continuing existing antiglaucoma medications. Postoperative pain can be effectively managed with oral paracetamol.

Several limitations of our study should be acknowledged. First, due to its retrospective nature, some data were missing, primarily because several patients were asleep during late-night measurement times, and a few paper-based records were lost. Second, treatment parameters varied slightly, with modifications applied in certain cases based on ocular status. Third, gaps between time points may have led to missed details about IOP trends. Fourth, the study included only patients with IOP records within the first 24 hours, as they were admitted for observation. This led to a smaller sample size and potential selection bias, excluding patients treated on an outpatient basis. Finally, patients in our study included various types of glaucoma, and most had severe or refractory diseases. Future prospective studies are needed to address these limitations.

## Conclusion

MP-TLT demonstrated an early and significant IOP reduction, beginning within the first hours after the procedure, with the maximum reduction observed at 1 week. While IOP and pain scores slightly increased at 5 hours compared to 1 hour, they gradually decreased over time. MP-TLT maintained a favorable IOP-lowering effect up to 12 months. However, its clinical success depends on preoperative IOP, target IOP, and individual treatment goals.

## Supporting information

S1 Table Subgroup analyses of intraocular pressure and pain during the first 24 hours postoperative period. (DOCX)
