# Supplementary material for: Intraocular pressure changes and pain scores within 24 hours and short-term outcomes after micropulse transscleral laser therapy
Source: PLoS One. 2026 Feb 6;21(2):e0340625. doi: 10.1371/journal.pone.0340625 (PMC12880698; doi:10.1371/journal.pone.0340625)
Supplement: S1 Table — (DOCX) [file pone.0340625.s001.docx]

**S1 Table. Subgroup Analyses of Intraocular Pressure and Pain During the First 24 Hours Postoperative Period.**

|  | **Preoperative** | **Postoperative** | | | | | |
| --- | --- | --- | --- | --- | --- | --- | --- |
|  |  | **Immediate** | **1 hour** | **5 hours** | **9 hours** | **13 hours** | **1 day** |
| **By baseline IOP** |  |  |  |  |  |  |  |
| ≤ 40 mmHg (21 eyes) |  |  |  |  |  |  |  |
| Mean IOP (SD) | 25.5 (9.2) |  | 24.0 (10.5) | 24.8 (11.9) | 21.5 (9.5) | 19.6 (8.9) | 15.4 (9.6) |
| *p-value* | *ref* |  | *0.476* | *0.997* | *0.105* | *0.043** | *<0.001** |
| Mean pain score (SD) |  | 2.5 (2.8) | 1.7 (2.0) | 2.3 (1.8) | 1.6 (1.7) | 1.3 (1.7) | 1.0 (1.5) |
| *p-value* |  | *ref* | *0.122* | *0.735* | *0.091* | *0.042** | *0.004** |
|  |  |  |  |  |  |  |  |
| > 40 mmHg (25 eyes) |  |  |  |  |  |  |  |
| Mean IOP (SD) | 53.2 (10.9) |  | 42.2 (17.1) | 45.2 (16.3) | 42.1 (15.2) | 39.3 (15.1) | 32.0 (13.8) |
| *p-value* | *ref* |  | *<0.001** | *<0.001** | *<0.001** | *<0.001** | *<0.001** |
| Mean pain score (SD) |  | 2.1 (3.0) | 3.0 (2.8) | 3.2 (2.6) | 1.7 (1.8) | 1.1 (1.5) | 0.9 (1.5) |
| *p-value* |  | *ref* | *0.085* | *0.028** | *0.429* | *0.153* | *0.019** |
|  |  |  |  |  |  |  |  |
| **By glaucoma diagnosis** |  |  |  |  |  |  |  |
| Primary glaucoma (11 eyes) |  |  |  |  |  |  |  |
| Mean IOP (SD) | 27.4 (14.3) |  | 22.8 (8.7) | 22.7 (8.7) | 21.7 (8.5) | 17.1 (7.9) | 14.4 (7.7) |
| *p-value* | *ref* |  | *0.092* | *0.082* | *0.034** | *<0.001** | *<0.001** |
| Mean pain score (SD) |  | 2.5 (2.4) | 1.8 (1.7) | 2.0 (1.2) | 1.7 (1.6) | 1.0 (1.4) | 0.4 (1.0) |
| *p-value* |  | *ref* | *0.268* | *0.417* | *0.213* | *0.022** | *0.001** |
|  |  |  |  |  |  |  |  |
| Secondary glaucoma (27 eyes) |  |  |  |  |  |  |  |
| Mean IOP (SD) | 46.7 (15.8) |  | 38.5 (18.2) | 44.3 (15.3) | 37.9 (16.4) | 37.2 (14.1) | 27.2 (14.4) |
| *p-value* | *ref* |  | *<0.001** | *0.246* | *<0.001** | *0.001** | *<0.001** |
| Mean pain score (SD) |  | 2.6 (3.2) | 2.5 (3.0) | 3.4 (2.4) | 1.9 (1.9) | 1.5 (1.8) | 1.2 (1.5) |
| *p-value* |  | *ref* | *0.819* | *0.075* | *0.219* | *0.18* | *0.007** |
|  |  |  |  |  |  |  |  |
| Childhood glaucoma (8 eyes) |  |  |  |  |  |  |  |
| Mean IOP (SD) | 38.0 (15.7) |  | 33.7 (15.8) | 25.8 (19.9) | 29.9 (19.8) | 27.0 (20.2) | 29.6 (17.3) |
| *p-value* | *ref* |  | *0.231* | *0.003** | *0.008** | *0.001** | *0.002** |
| Mean pain score (SD) |  | 0.5 (1.2) | 2.8 (1.7) | 1.4 (2.6) | 0.4 (0.5) | 0.3 (0.5) | 1.0 (2.0) |
| *p-value* |  | *ref* | *0.006** | *0.271* | *0.721* | *0.47* | *0.984* |
| *P-value <0.05  IOP indicates intraocular pressure; SD, standard deviation. | | | | | | | |
